# Analysis of maternal and neonatal outcomes using cervical cerclage or conservative treatment in singleton gestations with a sonographic short cervix

**DOI:** 10.1097/MD.0000000000025767

**Published:** 2021-05-07

**Authors:** Xiaoxiu Huang, Ruizhe Chen, Baohua Li

**Affiliations:** Department of Obstetrics, Women's Hospital, Zhejiang University School of Medicine, Hangzhou, Zhejiang, People's Republic of China.

**Keywords:** cervical cerclage, preterm birth, second trimester loss, short cervix length, sonographic diagnosis

## Abstract

To investigate the effect of cervical cerclage or conservative treatment on maternal and neonatal outcomes in singleton gestations with a sonographic short cervix, and further compare the relative treatment value.

A retrospective study was conducted among women with singleton gestations who had a short cervical length (<25 mm) determined by ultrasound during the period of 14 to 24 weeks’ gestation in our institution. We collected clinical data and grouped the patients according to a previous spontaneous preterm birth (PTB) at <34 weeks of gestation or second trimester loss (STL) and sub-grouped according to treatment option, further comparing the maternal and neonatal outcomes between different groups.

In the PTB or STL history cohort, the cerclage group had a later gestational age at delivery (35.3 ± 3.9 weeks vs 31.6 ± 6.7 weeks) and a lower rate of perinatal deaths (2% vs 29.3%) compared with the conservative treatment group. In the non-PTB-STL history cohort, the maternal and neonatal outcomes were not significantly different between the cerclage group and conservative treatment group. More importantly, for patients with a sonographic short cervix who received cervical cerclage, there was no significant difference in the maternal and neonatal outcomes between the non-PTB-STL group and PTB or STL group.

For singleton pregnant with a history of spontaneous PTB or STL and a short cervical length (<25 mm), cervical cerclage can significantly improve maternal and neonatal outcomes; however, conservative treatment (less invasive and expensive than cervical cerclage) was more suitable for those pregnant women without a previous PTB and STL history.

## 1 Introduction

Preterm birth (PTB) is the main cause of perinatal death, and ultrasound measurement of cervical length is one of the important clinical indicators for predicting PTB.^[1,2]^ The risk of delivering preterm is inverse to the length of the cervix. Prevention of PTB remains a challenge and there are numerous well-established maternal and fetal risk factors. Indeed, a cervical length <25 mm on ultrasound examination in the second trimester has been established as a risk factor for preterm delivery, but is not a specific marker predictive of cervical insufficiency.^[3–5]^

In recent decades, several non-surgical treatments and surgical modalities have been proposed to treat gravidas with a sonographic short cervix and a history of PTB or STL or without a history of PTB and STL. Although controversial,^[6–8]^ cervical cerclage is effective in the management of these patients, especially patients diagnosed with cervical insufficiency, according to the American College of Obstetrics and Gynecology (ACOG). Additionally, conservative treatments, including bed rest, vaginal pessary,^[9]^ and vaginal progesterone,^[10,11]^ may be effective in these patients, especially patients without a history of PTB or STL. At present, evidence is limited demonstrating the benefits of different treatments in women with a sonographic short cervix and a history of PTB or STL or without a history of PTB and STL. The study aimed to determine the effectiveness of cervical cerclage or conservative treatment on maternal and neonatal outcomes in these patients, and further compare the relative advantages.

## 2 Methods

**Figure 1:**
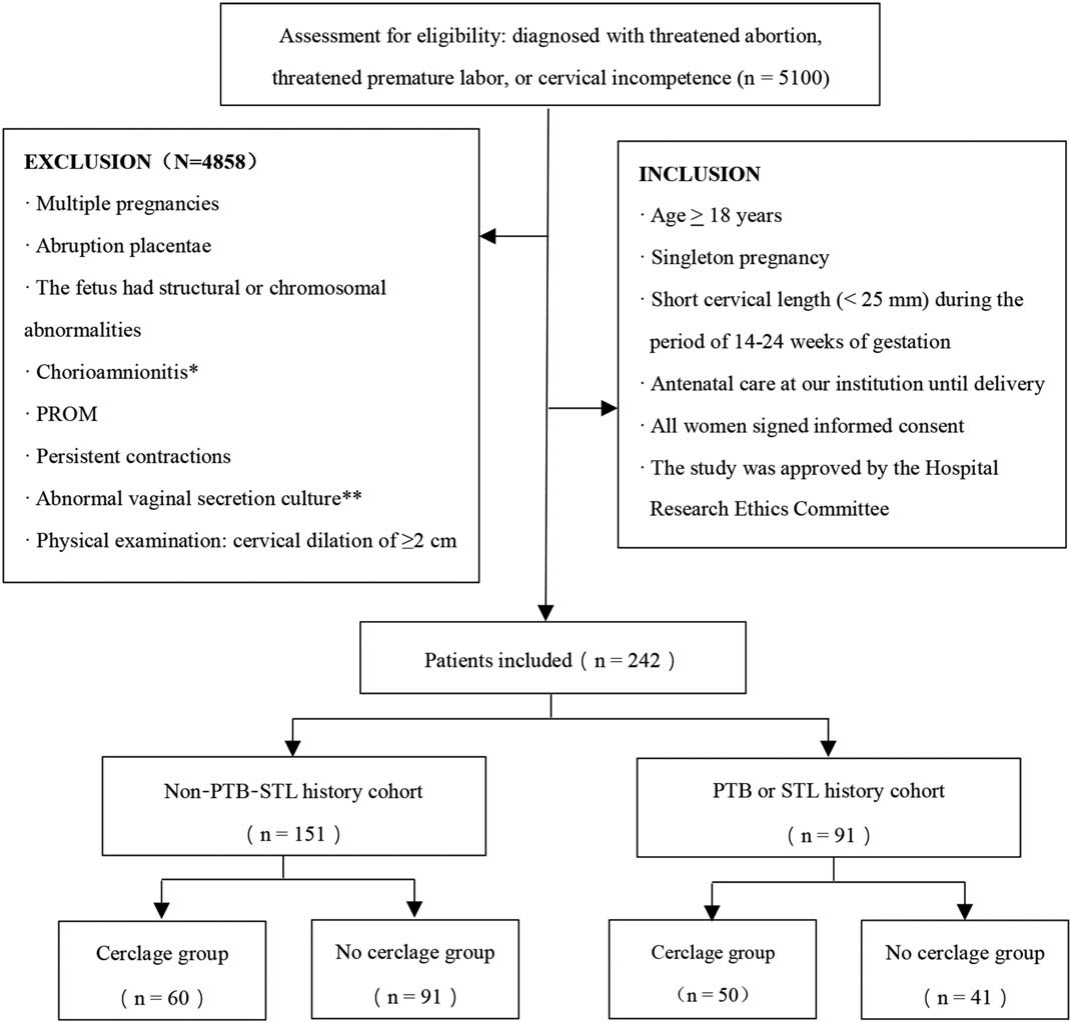
Flow diagram of the study. ^∗^Chorioamnionitis: uterine tenderness and/or temperature >38 °C or white blood cell (WBC) count >15 × 10^9^ L^−1^; ^∗∗^Abnormal vaginal secretion culture: Group B streptococcus-, mycoplasma-, chlamydia-, or bacterial culture-positive.

This retrospective cohort study enrolled pregnant women shown by ultrasound to have a short cervical length (<25 mm) during the period of 14 to 24 weeks of gestation from January 2004 to October 2018 in The Women's Hospital, Zhejiang University School of Medicine (Zhejiang, China). All patients were screened through review of the discharge diagnosis (including threatened abortion, threatened premature labor, or cervical incompetence) and met the inclusion and exclusion criteria (Fig. 1). The enrolled patients were delivered or aborted in our institution, grouped according to the presence of previous spontaneous PTB at <34 weeks of gestation or STL,^[12,13]^ and sub-grouped according to cervical cerclage (cerclage group) or conservative treatment (no cerclage group).

Cervical length was measured in the mid-sagittal plane by placing the calipers at each end of the endocervical canal, which was ascertained by transvaginal or transabdominal ultrasonography during the second trimester. The shortest cervical length was used for analysis when multiple cervical length measurements were obtained.^[14]^

The cervical cerclage procedure was performed under spinal anesthesia via the McDonald technique, which was performed transvaginally with a double “10” silk suture placed at the cervicovaginal junction without bladder mobilization.^[15]^ Perioperative management of patients with cerclage, such as the use of prophylactic antibiotics and/or bedrest and vaginal progesterone, was at the discretion of the clinicians. Cervical cerclages were removed at 36 to 37 weeks gestation with the exception of patients with ruptured membranes, the spontaneous onset of labor, or an indication for early delivery.

Conservative treatment included bed rest and vaginal progesterone. All women who received vaginal progesterone therapy were required to use progesterone soft capsules twice daily in the morning and at night (each pill contained 100 mg of progesterone; Besins Manufacturing Belglum, France).

We evaluated maternal demographic characteristics (including age, body mass index [BMI], gravidity, parity, reproductive history, prior conization, cervical length, maternal complications, and previous spontaneous PTB at ≤34 weeks of gestation or STL), current obstetric history, details of delivery, and neonatal outcomes (including birth weight, Apgar scores, perinatal deaths, the mean neonatal intensive care unit [NICU] stay, and neonatal complications). It should be noted that neonatal outcomes refer to live fetuses, with the exception of perinatal death.

Statistical analyses were performed using SPSS20.0 for Windows (SPSS Inc., Chicago, IL). Statistical significance was analyzed with a Chi-squared test or Fisher exact test for differences in qualitative variables and a *t* test for differences in continuous variables. A 2-sided alpha level <.05 was selected to represent statistical significance.

## 3 Results

### 3.1 Patient characteristics

A total of 242 pregnant women met the inclusion criteria of this study, including 151 pregnant women without a history of spontaneous PTB and STL, and 91 pregnant women with a history of spontaneous PTB or STL. In the PTB or STL history cohort (n = 91), 50 gravidas received cervical cerclage, and 41 gravidas received conservative treatment. In the non-PTB-STL history cohort (n = 151), 60 gravidas received cervical cerclage and 91 gravidas received conservative treatment. The demographic data of the 4 groups had no significant differences with respect to age, body mass index (BMI), parity, reproductive history, prior spontaneous PTB or STL, gestational age of prior PTB or STL, prior conization, cervical length, or maternal complications. Of note, gestational age at the treatment in the cerclage group (19.1 ± 4.1 weeks) was earlier in the PTB or STL history cohort than the other 3 groups (Table 1).

**Table 1:** The demographic characteristics of women with a short cervix (n = 242).

	Non-PTB-STL history cohort (n = 151)		PTB or STL history cohort (n = 91)	
Characteristics	No cerclage group (n = 91)	Cerclage group (n = 60)	No cerclage group (n = 41)	Cerclage group (n = 50)
Age, y^∗^	30.0 ± 4.4	31.2 ± 5.0	31.7 ± 5.3	30.7 ± 4.9
BMI, kg/m^2^^∗^	23.5 ± 4.2	24.4 ± 3.6	24.1 ± 2.7	23.3 ± 2.9
Gravidity^†^	1 (0–2)	1 (0–2)	2 (2–4)	2 (1–3)
Parity^†^	0 (0–1)	0 (0–1)	1 (0–1)	0 (0–1)
Reproductive history, n (%)				
Primiparous	64 (70.3)	41 (68.3)	20 (48.8)	30 (60.0)
Multiparous	27 (29.7)	19 (31.7)	21 (51.2)	20 (40.0)
Prior spontaneous PTB or STL^†^	–	–	1 (1–1)	1 (1–2)
Gestational age of prior PTB, wk^∗^	–	–	29.2 ± 1.8	28.4 ± 1.1
Gestational age of prior STL, wk^∗^	–	–	20.4 ± 4.2	20.8 ± 3.1
Prior conization, n (%)	2 (2.2)	5 (8.3)	2 (4.9)	2 (4.0)
Gestational age at the treatment, wk^∗^	23.0 ± 2.3	22.0 ± 3.9	23.0 ± 3.9	19.1 ± 4.1
Cervical length, mm^∗^	14.9 ± 7.1	14.1 ± 7.3	15.1 ± 7.7	17.3 ± 5.8
Maternal complications, n (%)	33	24	10	17
ICP	6 (6.6)	4 (6.7)	2 (4.9)	2 (4.0)
Thyroid hypofunction	6 (6.6)	3 (5.0)	2 (4.9)	4 (8.0)
Gestational diabetes mellitus	16 (17.6)	14 (23.3)	5 (12.2)	8 (16.0)
Gestational hypertension	3 (3.3)	2 (3.3)	1 (2.4)	2 (4.0)
Preeclampsia	2 (2.2)	1 (1.7)	0 (0.0)	1 (2.0)

BMI = body mass index, ICP = intrahepatic cholestasis of pregnancy, PTB = preterm birth, STL = second trimester loss.

∗ Mean ± SD.

† Median (range).

### 3.2 Maternal and neonatal outcomes in patients with a sonographic short cervix who received cervical cerclage or conservative treatment

In all enrolled patients, patients who received cervical cerclage did not differ from the no cerclage group with respect to the mean gestational age at delivery (34.8 ± 6.4 vs 35.6 ± 4.3 weeks; *P* > .05). The 2 groups did not differ significantly in the rate of preterm delivery or spontaneous preterm delivery at <34 and <37 weeks of gestation, but the rate of preterm birth or spontaneous preterm delivery at <28 weeks of gestation was lower in the women with a cerclage. The 2 groups did not differ significantly with respect to premature rupture of membranes (PROM), the birth weight of newborns, and Apgar scores (all *P* > .05; Supplemental Digital Content 1 [see Table, Supplemental Content, which demonstrates no difference in the maternal and neonatal outcomes in patients who received cervical cerclage or conservative treatment]).

In the PTB or STL history cohort, the cervical cerclage group was 35.3 ± 3.9 weeks’ gestation at delivery, which was significantly later than the no cerclage group (31.6 ± 6.7 weeks’ gestation, *P* < .01). The rate of preterm delivery at <28 (2%) and <34 weeks of gestation (36%) in the cervical cerclage group was significantly lower than the 31.7% and 58.5% observed in the no cerclage group (*P* < .01 and *P* < .05, respectively). Similarly, the cervical cerclage group had a lower rate of spontaneous preterm delivery at <28 and <34 weeks of gestation compared with the no cerclage group (*P* < .01 and *P* < .05, respectively). There were significant differences in the neonatal outcomes between the cerclage group and no cerclage group, such as greater birth weight of newborns (2549 g vs 1899 g; *P* < .01), lower birth weight <1500 g (14% vs 40%; *P* < .01), and the rate of perinatal deaths (2% vs 29.3%; *P* < .01). The 2 groups did not differ significantly in the rate of preterm delivery or spontaneous preterm delivery at <37 weeks of gestation, Apgar scores, PROM, mean NICU stay, and neonatal complications (all *P* > .05; Table 2).

**Table 2:** Maternal and neonatal outcomes in patients undergoing cervical cerclage or conservative treatment.

	Non-PTB-STL history cohort (n = 151)			PTB or STL history cohort (n = 91)		
	No cerclage group (n = 91)	Cerclage group (n = 60)	*P*	No cerclage group (n = 41)	Cerclage group (n = 50)	*P*
Gestational age at delivery, wk^∗^	36.2 ± 5.7	35.9 ± 4.6	.671	31.6 ± 6.7	35.3 ± 3.9	.002
Rate of preterm delivery, n (%)						
At <28 wk	11 (12.1)	4 (6.7)	.406	13 (31.7)	1 (2.0)	.000
At <34 wk	22 (24.2)	13 (21.7)	.844	24 (58.5)	18 (36.0)	.037
At <37 wk	29 (31.9)	24 (40.0)	.384	29 (70.7)	25 (50.0)	.055
Rate of spontaneous preterm delivery, n (%)						
At <28 wk	10 (11.0)	4 (6.7)	.568	13 (31.7)	1 (2.0)	.000
At <34 wk	21 (23.1)	12 (20.0)	.692	23 (56.1)	16 (32.0)	.033
At <37 wk	27 (29.7)	20 (33.3)	.720	27 (65.9)	23 (46.0)	.090
PROM, n (%)	22 (24.2)	13 (21.7)	.844	9 (22.0)	14 (28.0)	.629
PPROM	8 (8.8)	8 (13.3)	.424	5 (12.2)	11 (22.0)	.275
Term PROM	14 (15.4)	5 (8.3)	.223	4 (9.8)	3 (6.0)	.697
Perinatal deaths, n (%)	10 (11.0)	3 (5.0)	.247	12 (29.3)	1 (2.0)	.000
Birth weight, g^∗^	2738 ± 1009	2778 ± 938	.807	1899 ± 1125	2549 ± 805	.002
Birth weight <1500 g, n (%)	15 (16.5)	8 (13.3)	.650	16 (40.0)	7 (14.0)	.007
Apgar scores at 1 minute^∗^	9.86 ± 0.85	9.56 ± 1.23	.088	8.86 ± 2.17	9.02 ± 2.19	.757
Apgar scores at 5 minute^∗^	9.86 ± 1.01	9.88 ± 0.38	.926	9.59 ± 1.12	9.44 ± 1.80	.694
NICU admission, n (%)	11 (12.1)	14 (23.3)	.119	7 (17.1)	23 (46.0)	.060
Neonatal complications, n (%)						
Respiratory distress syndrome	7 (7.7)	7 (11.7)	.571	4 (9.8)	12 (24.0)	.387
Intraventricular hemorrhage	0 (0.0)	3 (5.0)	.068	1 (2.4)	4 (8.0)	.647
Suspected or proven early sepsis	2 (2.2)	3 (5.0)	.404	0 (0.0)	2 (4.0)	.529

PPROM = preterm premature rupture of membranes, PROM = premature rupture of membranes.

∗ Mean ± SD.

In the non-PTB-STL history cohort, there were no significant differences between the cerclage group and no cerclage group with respect to maternal outcomes, such as the mean gestational age at delivery, the rate of preterm delivery or spontaneous preterm delivery at <28, <34, and <37 weeks’ gestation, and PROM. With respect to the neonatal outcomes, the birth weight of newborns, Apgar scores, perinatal deaths, mean NICU stay, and neonatal complications were not significantly different between the 2 groups (all *P* > .05; Table 2).

### 3.3 Effect of cervical cerclage on maternal and neonatal outcomes in patients with a sonographic short cervix and a history of PTB or STL or without a history of PTB and STL

With respect to the sub-analysis of singleton pregnancies with placement of a cervical cerclage, there were no significant differences in the mean gestational age at delivery, the rate of preterm delivery or spontaneous preterm delivery at <28, <34, and <37 weeks’ gestation, PROM, mean birth weight, Apgar scores, perinatal deaths, and neonatal complications between the non-PTB-STL group (n = 60) and PTB or STL group (n = 50; *P* > .05; Table 3).

**Table 3:** Effect of cervical cerclage on maternal and neonatal outcomes in patients with a short cervix.

	Non-PTB-STL group (n = 60)	PTB or STL group (n = 50)	*P*
Gestational age at delivery, wk^∗^	35.9 ± 4.6	35.3 ± 3.9	.487
Rate of preterm delivery, n (%)			
At <28 wk	4 (6.7)	1 (2.0)	.374
At <34 wk	13 (21.7)	18 (36.0)	.136
At <37 wk	24 (40.0)	25 (50.0)	.338
Rate of spontaneous preterm delivery, n (%)			
At <28 wk	4 (6.7)	1 (2.0)	.374
At <34 wk	12 (20.0)	16 (32.0)	.189
At <37 wk	20 (33.3)	23 (46.0)	.168
PROM, n (%)	13 (21.7)	14 (28.0)	.508
PPROM	8 (13.3)	11 (22.0)	.312
Term PROM	5 (8.3)	3 (6.0)	.726
Perinatal deaths, n (%)	3 (5.0)	1 (2.0)	.624
Birth weight, g^∗^	2778 ± 938	2549 ± 805	.181
Birth weight, <1500 g, n (%)	8 (13.3)	7 (14.0)	.568
Apgar scores at 1 minute^∗^	9.56 ± 1.23	9.02 ± 2.19	.112
Apgar scores at 5 minutes^∗^	9.88 ± 0.38	9.44 ± 1.80	.760
NICU admission, n (%)	14 (23.3)	23 (46.0)	.025
Neonatal complications, n (%)			
Respiratory distress syndrome	7 (11.7)	12 (24.0)	.134
Intraventricular hemorrhage	3 (5.0)	4 (8.0)	.427
Suspected or proven early sepsis	3 (5.0)	2 (4.0)	.562

NICU = neonatal intensive care unit, PPROM = preterm premature rupture of membranes, PROM = premature rupture of membranes.

∗ Mean ± SD.

## 4 Discussion

Our study showed that among pregnant women with a sonographic short cervix and a previous spontaneous PTB or STL, cervical cerclage proved to afford more benefits with respect to maternal and neonatal outcomes than conservative treatment. The effects of cervical cerclage and conservative treatment for patients without a history of spontaneous PTB and STL were similar. These results suggested that conservative treatment may provide a benefit for women with a short cervix but without a history of PTB and STL.

Several studies have confirmed that a cervical length <25 mm on ultrasound examination in the second trimester is a powerful predictor of preterm delivery in patients with a history of PTB or STL or without a history of PTB and STL.^[3,4,16,17]^ Based on a review of the literature, very few studies have systematically investigated the indications for conservative treatment or cervical cerclage among patients with a sonographic short cervix (<25 mm).^[9,18–23]^ In the To et al^[18]^ study, 253 women with a short cervical length (≤15 mm) were measured by transvaginal ultrasound between the 22 and 24 weeks gestation agreed to participate in a randomized study of cervical cerclage. The rate of preterm delivery at <33 weeks gestation was similar between the cerclage group (22%) and no cerclage group (26%; relative risk = 0.84, 95% CI 0.54–1.31, *P* = .44). Rust et al^[23]^ randomized 61 patients with a cervical length <25 mm or prolapse of the fetal membranes into the endocervical canal ≥25% of the original cervical length to receive cervical cerclage or no cerclage. There was no difference in gestational age at delivery between the cerclage group (33.8 ± 6.0 weeks) and no cerclage group (33.8 ± 5.5 weeks). Our results confirmed that there was no significant difference in the mean gestational age at delivery between the cerclage group and no cerclage group (34.8 ± 6.4 vs 35.6 ± 4.3 weeks, respectively). Cerclage was not associated with a significant reduction in the rate of preterm delivery at <34 weeks’ gestation compared with conservative treatment. In the trial conducted by To et al or Rust et al, the enrolled patients included patients with a history of PTB or STL or without a history of PTB and STL and included singleton and multiple pregnancies. But they did not indicate the number of patients with a history of PTB or STL and without a history of PTB and STL. In contrast with the articles described above, we grouped the patients by a previous spontaneous preterm birth (PTB) at ≤34 weeks of gestation or second trimester loss (STL) and sub-grouped by treatment option according to ACOG guidelines. The safety and efficacy of cervical cerclage and conservative treatment on maternal and neonatal outcomes were further compared. Data from our study may be helpful in providing optimal treatment on the application of ACOG guidelines in patients with a short cervix.

According to ACOG and RCOG guidelines, cervical cerclage often is recommended for women who are diagnosed of cervical insufficiency with a prior spontaneous PTB before 34 weeks gestation, and a short cervical length (<25 mm) before 24 weeks gestation (also known as ultrasound-indicated cerclage). In the randomized trial by Althuisius et al,^[19]^ 30 patients had a cervical length <25 mm measured before 24 weeks gestation. The cerclage group had a later mean gestational age at delivery compared with the bed rest group (37.9 ± 1.3 vs 32.4 ± 6.5 weeks, respectively). Wang et al^[20]^ further demonstrated that delivery ≥37 weeks of gestation in the cervical cerclage group (63.4%) was higher than the vaginal progesterone group (33.3%). However, Berghella et al^[24]^ reported that among singleton gestations with a cervical length <25 mm and a previous PTB <35 weeks of gestation there was no differences in PTB <35 weeks of gestation between the cerclage group (40%) and no cerclage group (56%). Our data confirmed that patients with a sonographic short cervix and a history of PTB or STL who underwent cervical cerclage had a more advanced gestational age at delivery and a lower PTB rate before 34 weeks’ gestation compared with conservative treatment (36% vs 58.5%, respectively). Moreover, we analyzed the neonatal outcomes between the 2 groups and found that the rate of birth weights <1500 g (14%) and perinatal deaths (2%) in patients with cervical cerclage were lower than conservative treatment in this subgroup, which was in agreement with another report in the literature.^[20]^ Our results suggest that cervical cerclage can significantly improve maternal and neonatal outcomes for singleton gestations with a sonographic short cervix and a history of spontaneous PTB or STL.

The safety and efficacy of cervical cerclage in women with a short cervix (<25 mm), but without a history of PTB and STL have not been fully elucidated. Parrish et al^[25]^ retrospectively analyzed 70 nulliparous women with a cervical length <25 mm and found that the risk of delivering preterm at <32 weeks gestation was increased in the cerclage group when compared with the expectantly managed group (RR = 6.7; 95% CI = 1.45–30.6). However, Wang et al^[20]^ reported that there were no significant differences in the proportion of delivery ≥37 weeks of gestation between the cervical cerclage group and vaginal progesterone group (55.9% vs 60.9%, respectively). Our results suggest that in this subset of patients, although the clinical characteristics of these groups were basically the same, cervical cerclage would not be beneficial in preventing PTB in women without a history of PTB and STL. The mean gestational age at delivery and the rate of preterm delivery at <28, <34, and <37 weeks of gestation did not differ significantly between the cerclage group and conservative treatment group. It was noted that patients undergoing cerclage placement had an earlier gestation at delivery than those managed without cerclage (35.9 ± 4.6 vs 36.2 ± 5.7 weeks; *P* > .05). It is possible that cervical cerclage may induce a local inflammatory reaction and preterm premature rupture of membranes in these patients.^[26]^ Although our data have confirmed that cerclage is safe, conservative treatment is more suitable for singleton gestations with a sonographic short cervix but without a previous spontaneous PTB and STL because conservative treatment is less invasive and costly.

The efficacy of cervical cerclage in the treatment of a short cervical length (<25 mm) with a history of PTB or STL or without a history of PTB and STL was further evaluated in our study. We performed cervical cerclage in these patients, and analyzed the maternal and neonatal outcomes between the non-PTB-STL group and PTB or STL group. Our results showed that there was no significant difference in the mean gestational age at delivery, the rate of preterm delivery at <28, <34, and <37 weeks of gestation, and the perinatal deaths rates between 2 groups in patients who received cervical cerclage. This data showed that cervical cerclage has more benefits with respect to the maternal and neonatal outcomes for patients with a history of PTB or STL, and had the same effect as patients without a history of PTB and STL. The results herein provide additional evidence for the application of ACOG guidelines in making a decision to place a cervical cerclage in women at risk of or diagnosed with cervical insufficiency.

The relatively small number of patients was a limitation of this retrospective cohort study. Another limitation was the absence of randomized cohorts, and the choice of treatment options should have taken into account the desires of the patients’ and recommendations of the clinicians’. A further study with a larger number of patients is thus warranted. We will compare cervical cerclage and conservative treatment in an adequately powered randomized trial involving patients with a history of PTB or STL or without a history of PTB and STL.

## 5 Conclusions

Our data suggest that the indications for conservative treatment or cervical cerclage among women with singleton gestations and a sonographic short cervix are different. In pregnant women with a short cervical length (<25 mm) and a previous spontaneous PTB or STL, cerclage was safer and more effective, while conservative treatment was more suitable for patients without a previous spontaneous PTB and STL because conservative treatment is less invasive and expensive. Nevertheless, randomized controlled trails should be conducted to confirm our conclusion.

## Acknowledgments

The authors thank the pregnant women for their participation in our study.

## Author contributions

**Data curation:** Xiao-Xiu Huang, Rui-Zhe Chen.

**Formal analysis:** Xiao-Xiu Huang.

**Methodology:** Bao-Hua Li.

**Supervision:** Bao-Hua Li.

**Writing – original draft:** Xiao-Xiu Huang.

**Writing – review & editing:** Bao-Hua Li.

## Supplementary Material

Supplemental Digital Content
