# Supplementary material for: Analysis of maternal and neonatal outcomes using cervical cerclage or conservative treatment in singleton gestations with a sonographic short cervix
Source: Medicine (Baltimore). 2021 May 7;100(18):e25767. doi: 10.1097/MD.0000000000025767 (PMC8104303; doi:10.1097/MD.0000000000025767)
Supplement: Supplemental Digital Content [file medi-100-e25767-s001.doc]

**Supplemental Digital Content 1:** **Maternal and neonatal outcomes in patients undergoing cervical cerclage or conservative treatment**

|  | **No cerclage group**  **(n = 132)** | **Cerclage group**  **(n = 110)** | ***P*** |
| --- | --- | --- | --- |
| Gestational age at delivery (wk)* | 34.8±6.4 | 35.6±4.3 | 0.267 |
| Rate of preterm delivery, n (%) |  |  |  |
| At＜28 wk, | 24（18.2） | 5（4.5） | 0.001 |
| At＜34 wk, | 46（34.8） | 31（28.2） | 0.332 |
| At＜37 wk,  Rate of spontaneous preterm delivery, n (%)  At＜28 wk  At＜34 wk,  At＜37 wk | 58（43.9）  23（17.4）  44（33.3）  54（40.9） | 49（44.5）  5（4.5）  28（25.5）  43（39.1） | 1.000  0.002  0.205  0.694 |
| PROM, n (%)  PPROM  Term PROM | 31（23.5）  13（9.8）  18（13.6） | 27（24.5）  19（17.3）  8（7.3） | 0.880  0.126  0.145 |
| Perinatal deaths, n (%) | 22（16.7） | 4（3.6） | 0.001 |
| Birth weight (g)* | 2477±1112 | 2675±884 | 0.133 |
| Birth weight <1500 g, n (%) | 31（23.5） | 15（13.6） | 0.300 |
| Apgar scores at 1 minute* | 9.60±1.39 | 9.31±1.76 | 0.176 |
| Apgar scores at 5 minute* | 9.79±1.04 | 9.67±1.27 | 0.455 |
| NICU admission, n (%) | 18（13.6） | 37（33.6） | 0.003 |
| Neonatal complications, n (%) | | | |
| Respiratory distress syndrome | 11（8.3） | 19（17.3） | 0.117 |
| Intraventricular hemorrhage | 1（0.8） | 7（6.4） | 0.034 |
| Suspected or proven early sepsis | 2（1.5） | 5（4.5） | 0.275 |

*Mean ± SD; PROM: premature rupture of membranes; PPROM: preterm premature rupture of membranes

Article title: Analysis of maternal and neonatal outcomes using cervical cerclage or conservative treatment in singleton gestations with a sonographic short cervix

First author: Xiaoxiu Huang MD
